# The relationships between sexual dysfunctions, psychopathology and treatment in patients with schizophrenia

**DOI:** 10.1192/j.eurpsy.2023.338

**Published:** 2023-07-19

**Authors:** A. Bejar, N. Smaoui, R. Feki, I. Gassara, S. Omri, N. Charfi, J. Ben Thabet, L. Zouari, M. Maalej Bouali, M. Maalej

**Affiliations:** psychiatry “C”, Hedi Chaker university hospital, Sfax, Tunisia

## Abstract

**Introduction:**

Sexual dysfunctions (SD) are common in patients with schizophrenia. The link between schizophrenia and sexuality is complex. Studies have shown that SD can be linked to the side effects of antipsychotic medications, but also to symptoms of illness.

**Objectives:**

To identify the clinical and therapeutic factors associated with SD in outpatients with schizophrenia.

**Methods:**

A cross-sectional and analytical study was conducted between Mars and September 2019. It included 53 outpatients with schizophrenia in clinical remission for at least two months.

We used the Positive and Negative Symptom Scale (PANSS) to assess clinical symptoms and the Arizona Sexual Experiences Scale (ASEX) to assess sexual functioning.

**Results:**

The average age was 42.28 ±10.49 years old. The sex ratio was 3.81. The mean age of onset was 27.09±5,46 years. The mean duration of illness was 18.11±9.29 years. First-generation antipsychotics were prescribed in 77.4% of cases, while second-generation antipsychotics were prescribed in 39.6% of cases.

The average ASEX score was 19.77±5.99, and 67.9% of participants had at least one SD. the analytical study revealed significantly higher average scores for the PANSS-negative subscale (p=0.006) and the PANSS total score (p=0.04) in patients with SD. SD correlated with first-generation antipsychotic treatments (p=0.02).

**Conclusions:**

Our results show that SD are frequent in patients with schizophrenia and that they are related to the severity of the symptoms, in particular the negative symptoms of illness, and the prescription of first-generation antipsychotics.

**Disclosure of Interest:**

None Declared

